# Exploring deep brain stimulation willingness in Black individuals with Parkinson’s disease

**DOI:** 10.1016/j.neuros.2025.100012

**Published:** 2025-09-29

**Authors:** Chiamaka Onuigbo, Chantale Branson

**Affiliations:** a Emory School of Medicine, 100 Woodruff Circle, Atlanta, GA, 30322, USA; b Morehouse School of Medicine, 720 Westview Dr. SW, Atlanta, GA 30310, USA

**Keywords:** Parkinson’s disease, Health disparities, Deep brain stimulation, African American populations, Patient perspectives, Treatment access

## Abstract

**Background:**

Black individuals with Parkinson’s disease (PD) receive deep brain stimulation (DBS) at significantly lower rates than non-White Hispanic individuals, a disparity associated with poorer outcomes.

**Methods:**

To explore potential patient-level factors contributing to decreased utilization, we conducted a cross-sectional pilot survey among 28 Black adults with PD who had not undergone DBS. Participants were recruited from neurology clinics in an urban safety-net hospital and local support groups in Atlanta. A structured survey was given to assess views towards DBS, including willingness to undergo DBS both currently and in a hypothetical scenario of worsening symptoms. Responses (YES, NO, MAYBE) were dichotomized (YES vs NO/MAYBE) for analysis. Thematic analysis explored participant perceptions.

**Results:**

The median age was 73.5 years. 50 % were male. When asked about current consideration of DBS, 8 (29 %) said YES, 14 % (50 %) said NO, and 6 (21 %) said MAYBE. In the hypothetical scenario, 19 (68 %) said YES, 4 (14 %) said NO, and 5 (18 %) said MAYBE. Notably, 12 participants shifted from NO/MAYBE to YES, while only one shifted in the opposite direction, indicating a significant increase in willingness under worsening conditions (X^2^ = 7.692, p = 0.01). The most common reasons for hesitation were surgical concerns and lack of information.

**Conclusion:**

Findings suggest that rather than patient reluctance, reduced DBS uptake in this population may be more reflective of modifiable systemic barriers, such as limited awareness or disparities in the referral process. Educational outreach and equitable access to advanced therapies may address this gap.

**Clinical trial statement:**

This was a survey study and not a randomized clinical trial, so there is no clinical trial registration number.

## Introduction

Parkinson’s disease (PD) is a progressive neurodegenerative condition, with an estimated 60,000 to 95,000 new diagnoses each year [1]. As PD progresses, patients experience motor fluctuations, dyskinesias, and non-motor disabilities, all with a narrowing therapeutic window in dopaminergic therapy [2]. Deep brain stimulation (DBS) has emerged as the gold standard intervention for advanced PD, resulting in better motor symptom control compared to optimized medical therapy [3]. In the context of PD, DBS is typically utilized in levodopa-responsive patients who have suboptimal control with their current medication regimen, such as medication intolerance, bothersome dyskinesias, decreasing medication efficacy, or medication refractory tremor [4–6]. Despite increased use over the years, there remains disparate access in different populations [7,8]. For instance, Black patients are 5–8 times less likely than White patients to undergo DBS implantation [9–11]. Even after adjusting for variables such as lower socioeconomic status (SES), age, and insurance type, the disparities persist [8,9,11], suggesting that additional barriers, including social determinants of health (SDoH), affect access in underserved populations. Continued investigation of these barriers is essential in addressing poorer health outcomes observed among marginalized groups, while guiding efforts to achieve more equitable care in PD.

Understanding patient-centered barriers to DBS can help explain underutilization in certain populations. While PD patients recognize the potential of DBS to improve symptoms, their hesitations center on efficacy, surgical concerns, costs, complications, and the social stigma regarding implantable therapy [12–15]. However, the studies’ populations that produced these revelations were predominantly White or college educated, limiting insight into minority patients’ opinions. In studies regarding persistent disparities in DBS use, authors proposed that underutilization in minority groups could be due to mistrust of the healthcare system [16,17], different health seeking behaviors [18], cultural biases against device aided therapies [9], and reduced patient engagement in their care [17]. Of note, these inferences were derived from behaviors seen in other disease states. Given the limited information regarding minority patients’ beliefs about DBS, our study aims to assess the willingness of Black PD patients to undergo DBS, by collecting survey responses from patients regarding their perceptions and awareness of this procedure. The information gleaned from this study will generate insights into patient driven factors that may contribute to lower utilization.

## Materials and methods

This survey study enrolled patients with PD in the Atlanta metropolitan area who self-identified as Black. Patients were recruited from general and movement disorder neurology clinics at Grady Memorial Hospital (GMH) and from local support group meetings organized by American Parkinson’s Disease Association (APDA). Exclusion criteria included minors, individuals with cognitive or psychiatric conditions that impaired their ability to participate in informed consent discussions, those with DBS implants, and nonfluent English speakers. Any clinic participant with an ICD-10 code for any type of dementia were excluded from this study, and the physician or support group leader’s discretion was used when assessing which individuals could participate in informed consent discussions, given any other comorbidities. This study was approved by the Emory University Institutional Review Board (IRB), Morehouse School of Medicine IRB, and Grady research oversight committee (protocol number STUDY00006870). Participants provided signed informed consent for in-person visits or verbal informed consent for telephone visits prior to enrollment.

Potential clinic participants were prospectively identified through a registry of patients who attended a neurology clinic at GMH, with an ICD-10 code corresponding to a diagnosis of PD. Additionally, the EMR was used to identify patient’s race. If race was not listed, participants were approached and asked to verbally confirm their race before participating in the study. Consequently, all clinic individuals who agreed to participate were patients of the movement disorders clinic. After obtaining approval from the APDA executive director and chapter coordinator, local support group leaders were contacted and asked if 1) the survey could be administered at the end of a meeting, and 2) if they had attendees who identified as Black. If the support group leaders agreed to survey administration and had Black participants, then a research team member administered the paper survey in person at the end of the meeting for interested participants. Support group participants had to verbally self-identify as Black and confirm a diagnosis of PD before completing the survey. Participants were given the option for the researcher to read the questions for them and record their answers, or participants could answer the questions on their own, with the research member close by for any needed clarification of questions. Participants who expressed interest but could not complete the survey at that time had the option to provide a phone number to complete the survey over the phone. In this instance, the questions would be read over the phone, and the researcher would record the answers given by the participant on the paper form. The primary outcome of the study was the assessment of willingness to undergo DBS, and the secondary outcome was the evaluation of perceptions regarding DBS.

All researchers had to follow the same verbal script while presenting the survey, and participants completed a 12- question survey divided into three sections (Supplementary Fig. 1). All responses were free-text, except for one, which used a 5-point Likert scale to assess the perceived disability of symptoms (1= not disabling at all; 5 = extremely disabling). The first section collected demographic information, including age, gender, education level, and time since diagnosis. If participants were not able to recall the month and year of diagnosis, they were encouraged to give their best estimate. A clinical chart review was conducted on participants with established records at GMH to confirm the diagnosis date. In cases of discrepancy between the two dates, the one documented in the chart was used in analysis. The second section included questions about quality of life (QoL), such as the most bothersome symptom and medication regimen. The third section addressed awareness of alternative PD treatments and willingness to consider DBS under two different scenarios, accompanied by brief explanations for their choices. Before answering the two DBS- related questions, participants were provided a standardized, short description outlining what DBS involves, its indications, and potential risks and benefits.

Descriptive statistics were used to summarize the demographic and clinical characteristics of the study population, with medians and interquartile ranges (IQRs) as appropriate. Frequencies and percentages were reported for categorical variables. For free-response categorical answers, the biostatistician and first author independently reviewed responses to identify recurrent themes and coded them emergent categories. For vague responses, they used their best judgement to assign responses to the appropriate categories, discussing discrepancies in assignment until a consensus was achieved. Responses that did not fit the emergent themes, or responses that were only listed once, were placed into the “other” category. This delineation allowed for consistent classification of free-text responses while maintaining fidelity to the participants’ responses.

Associations between categorical variables were assessed using Fisher’s exact test, while the Wilcoxon rank-sum test was employed for comparisons of continuous variables between two groups. To assess within-subject changes in DBS acceptance between current and future scenarios, we used McNemar’s exact test with an Edward’s correction for paired binary data. Analyses examined factors associated with awareness of and willingness to undergo DBS, including demographic variables, symptom severity, medication burden, and prior discussion of alternative treatment options. All tests were performed in a two-sided manner, and statistical significance was set at p < 0.05. All analyses were performed using SAS version 9.4 (SAS Institute, Cary, NC, USA).

Due to the small sample size, “maybe” and “no” responses were combined and compared to “yes” responses to strengthen statistical power. Sensitivity analyses were performed post hoc to assess the robustness of the primary outcome categorization. These included two alternative comparisons: 1) grouping “maybe” with “yes” and comparing with “no” (YES/MAYBE vs NO), and 2) excluding “maybe” responses and analyzing only “yes” versus “no” trends (YES vs NO). These analyses aimed to determine whether the findings remained consistent across different categorizations.

## Results

Participants were recruited from January 2024 to June 2024. Fifty-one participants were eligible and 28 agreed to participate in this study. Eight patients did not show up to clinic, 5 declined to participate, and 10 participants did not answer the phone after the visit. The median age was 73.5 years old and 50 % (n=14/28) completed high school or equivalent. The median time from initial diagnosis was 24 months (IQR = 8.5, 36). Most participants reported moderate to very disabling symptoms, with a median rating of 4 out of 5 on the Likert scale (IQR = 3, 4.5). The most troublesome symptom was tremor (46 %, n =13/28). The “other” category listed in Table 1 consisted of balance difficulties, thermal dysregulation, REM sleep behavioral disorder, and blood pressure fluctuations. The median number of PD medications taken was 1 (IQR = 0.5, 1.5), and the median frequency of medication use was 3 times a day (IQR = 1.5, 3.5). Table 1 contains the remaining demographic information.

### Treatment awareness

Given varying reports of DBS awareness in literature, we sought to assess degree of awareness of parkinsonian treatment. Most of the participants, 68 % (n = 19/28), were not aware of treatment options that were different from their current medication regimen, and 68 % had not heard about DBS prior to this study (Table 1). Increased DBS awareness was associated with knowledge of other treatment options (p = 0.013). Among participants who were aware of DBS, 67 % (n = 6/9) also reported knowledge of additional treatments, whereas only 16 % (n = 3/16) of those unaware of DBS indicated such knowledge. Other factors such as age, gender, level of education, most troublesome symptom, time spent living with the disease, perceived disease severity, frequency of medication taken, or the number of different medications taken were not associated with DBS awareness (Table 2).

### DBS willingness

**Fig. 1.**
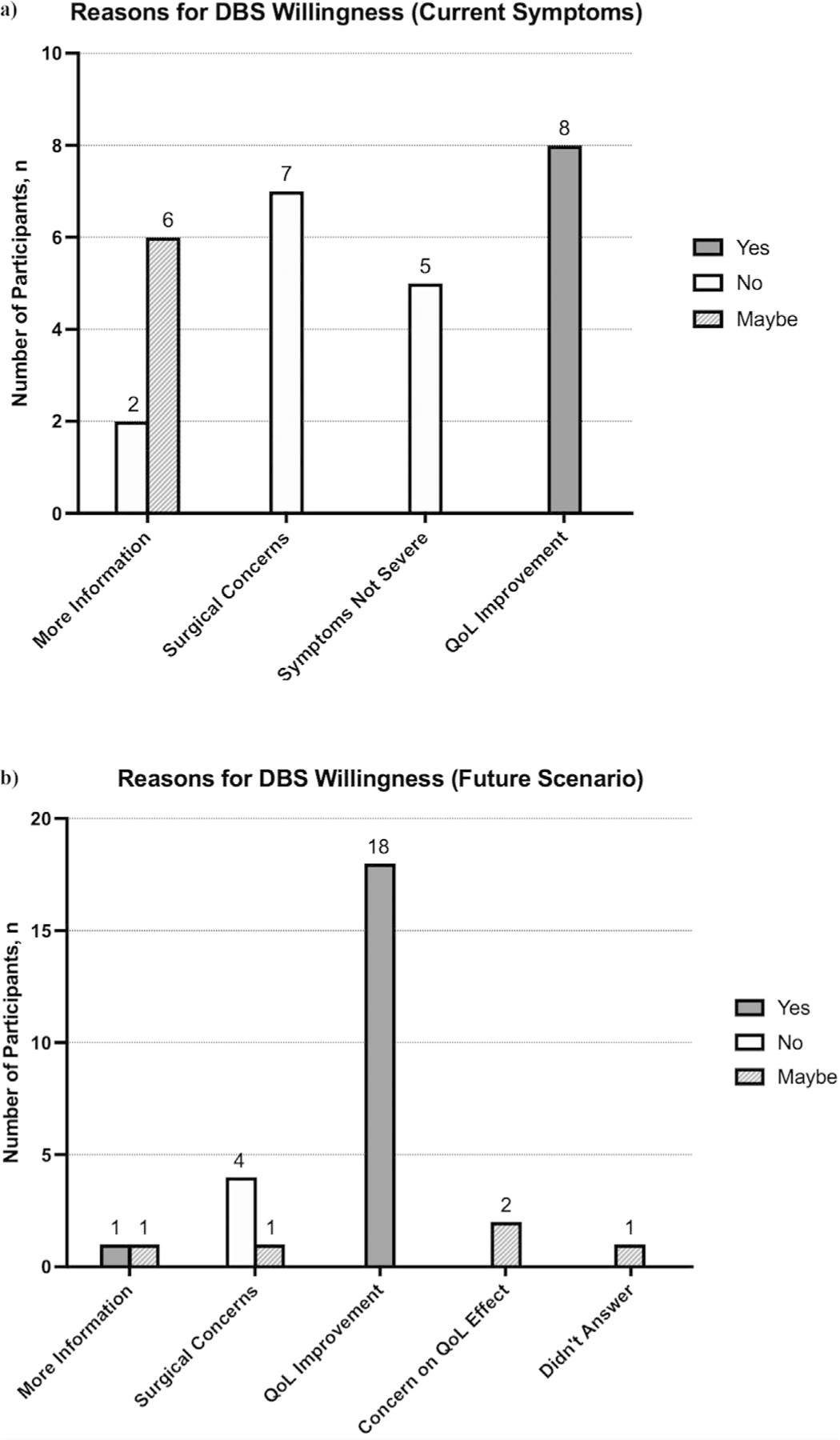
Patient-reported reasons for willingness to undergo DBS in two scenarios. (a) Reasons given after participants answered the question “Would you undergo DBS today?”. (b) Reasons given after participants answered the question “Would you consider undergoing DBS in the future if your symptoms were very disabling despite maximum medical therapy?”. Abbreviations: DBS, deep brain stimulation; QoL, quality of life.

To assess willingness, we asked participants whether they would consider DBS as a treatment option, given their current symptoms. Eight (29 %) participants said YES, 14 (50 %) said NO, and 6 (21 %) said MAYBE. The top reason for YES was potential improvement of QoL, for MAYBE was a desire for more information, and for NO was surgical concerns. Fig. 1a displays the complete set of responses. Current DBS willingness was associated with male gender (p = 0.03), greater perceived disability (p = 0.03) and increased number of daily medications (p = 0.04). Other factors such as age, education level, awareness of other treatments, most bothersome symptom, disease duration, and frequency of daily medication use were not statistically significant (Table 3).

We also asked participants a hypothetical scenario to assess consideration of DBS if symptoms were very disabling despite optimization of medical management. Nineteen (68 %) participants said YES, 4 (14 %) said NO, and 5 (18 %) said MAYBE. The top reason for YES was potential to improve QoL, for MAYBE was uncertainty about effects on QoL, and for NO was surgical concerns. Fig. 1b displays the complete set of responses for the hypothetical scenario. Factors associated with future DBS willingness were increased daily number of medications (p = 0.002) and increased daily medication frequency (p = 0.005). Age, gender, disease duration, symptom type, education level, awareness of other treatments, and perceived disability were not associated with future DBS willingness (Table 4).

Among the 28 participants, 12 shifted from NO/MAYBE to YES when asked about future willingness to consider DBS, while only 1 shifted from YES to NO/MAYBE, indicating a significant increase in willingness under the hypothetical scenario (X^2^ = 7.692, p = 0.01). This indicates that in the hypothetical scenario, patients are more willing to undergo DBS compared to their current state.

### Sensitivity analyses results

We performed sensitivity analyses to assess the impact of alternative groupings on associated factors. In the YES/MAYBE vs NO recategorization, younger age (p = 0.02), higher education level (p = 0.03), and increased symptom severity (p = 0.03) were associated with current DBS willingness (Supplementary Table 1). Younger age (p = 0.03) increased daily medication frequency (p = 0.02), and increased daily medications (p = 0.01) were associated with future DBS willingness (Supplementary Table 2). When comparing responses between current and future DBS willingness, 10 participants changed from NO to YES/MAYBE, while no participant changed from YES/MAYBE to NO (X^2^ = 8.1, p = 0.004).

In the YES vs NO categorization, 20 participants answered either YES or NO regarding willingness to undergo DBS currently and 23 participants for willingness to undergo DBS in the future. The significant variables for current DBS willingness include male gender (p = 0.03) and increased symptom severity (p = 0.02) (Supplementary Table 3). The variables associated with future DBS willingness include younger age (p = 0.04), increased medication frequency (p = 0.01), and increased number of medications (p = 0.01) (Supplementary Table 4). When comparing responses to current and future willingness, 8 participants changed from NO to YES while no participant changed from YES to NO (X^2^ = 6.125, p = 0.01).

## Discussion

Participants demonstrated greater willingness to consider DBS when presented with a hypothetical scenario of a more advanced disease state, compared to their current situation. The most cited reasons for hesitation include surgical concerns, wanting more information, and lack of severe symptoms. Baseline awareness of DBS in this population was low.

In a study on perceptions of earlier use of DBS, no participants wanted DBS surgery at that time, despite suboptimal symptom control and high medication burden, yet they were open to it in the future [19]. This is similar to our findings, where only 29 % of participants expressed current willingness to undergo DBS. Additionally, our findings showed significantly increased future DBS willingness if symptoms became disabling despite optimal medical therapy, a persistent trend in the post-hoc sensitivity analyses. This suggests that initial reluctance may stem more from perceived lack of necessity than from outright rejection of advanced therapies, refuting claims that Black people are less receptive to procedures [16,20–22]. Instead, responses revealed the value that participants place on maintaining QoL [23], even with limited knowledge of DBS risks and benefits, as worse perceived disability and increased medication burden were significantly associated with current and future willingness to consider DBS.

Male gender was significantly associated with willingness to undergo DBS given current symptoms, and the same trend, although not statistically significant, was seen for future willingness. However, gender was not a significant variable in post-hoc sensitivity analyses. Although prior research suggests that decreased implantation rates in women might stem from stronger personal preferences or increased caution against surgical procedures [15,24], our findings reveal that the personal differences in Black men and women might not affect DBS willingness.

Only 32 % of participants had prior knowledge of DBS. Awareness was significantly associated with having heard of other PD treatments, pointing to a gap in patient education and access to information [25]. This may contribute to lower DBS utilization rates in racial minorities, despite a relatively high willingness to consider the procedure in the future. Previous studies show that patients with greater awareness of DBS are often those who engage more with structured educational resources outside of routine clinical care, such as programs offered by nonprofit organizations focused on PD [19,26,27]. Although this study did not assess health-seeking behaviors for sources of information, the low awareness levels might reveal limited exposure to PD specific educational programming hosted by nonprofit organizations. This hypothesis is also supported by the fact that survey studies using PD-specific nonprofit participants have demographic characteristics that are overwhelmingly White and highly educated [12,15]. We observed similar patterns during recruitment for support groups, where most had no Black participants and those that did had only a small minority. This educational gap may reflect systemic inequities in how health information is spread and which communities are reached. Future efforts in outreach events that target underrepresented groups can improve equity in knowledge and access to therapies, while ameliorating the effects that a shortage of movement disorder specialists can have in equal access to care.

Importantly, most participants in our study would not yet qualify for DBS under current guidelines, which recommend waiting at least 4 years after diagnosis [28,29]. Also, many had not utilized alternative oral options for symptom control. This may partially explain low DBS awareness, as discussions about advanced therapies are usually initiated when a provider deems a patient as eligible [30]. Such low awareness places more onus on providers to initiate conversations and determine candidacy. When these decisions are left largely to clinical judgement, implicit biases may play a great role, particularly in the absence of structured referral pathways to DBS [31,32]. One way to ameliorate low levels of awareness is by normalizing early conversations surrounding DBS and other advanced therapies, despite eligibility status. This proactive approach empowers patients to explore options, address modifiable barriers affecting candidacy, and promotes transparency in clinical decision-making [33,34]. This can also promote open dialogue and shared decision making between patient and provider, allowing for periodic re-evaluation of individual patient situations [35,36] while ensuring that their values and preferences are at the forefront of joint decision making [37].

The top reasons for reluctance toward DBS included surgical concerns, wanting more information, and lack of severe symptoms. In both immediate and future contexts, surgical hesitancy remained prominent, highlighting the influence of negativity bias in shaping attitudes [38,39]. These findings underscore the need for ongoing, accessible education about DBS. Physician-led discussions are often limited by time constraints and infrequent clinic visits, which might be insufficient as DBS misconceptions persist among those who believe they are well-informed [40–42]. To address this, longitudinal discussions and referrals to external sources, like patient advocates, support groups, and educational materials centered on DBS can help dispel misunderstandings and decrease patient reluctance [43–46]. As people’s circumstances and symptoms evolve, so do their perspectives. Ongoing dialogue not only manages expectations but facilitates informed future decision-making [47–49]. By shaping attitude towards DBS, it may ultimately improve willingness to pursue the procedure [50].

This study has several limitations. As a pilot with a small sample size, it was underpowered to detect statistically significant differences, and no effect size was calculated. The lack of a control group, such as White PD participants, limits the ability to infer if observed trends are specific to Black participants of if they reflect broader disparities in DBS awareness and acceptance. Despite these constraints, these findings provide important preliminary insights into patient-centered factors for decreased utilization in Black individuals. Sensitivity analyses showed consistent trends, especially regarding increased future willingness to undergo DBS if deemed necessary, supporting the credibility of the exploratory results. The hypotheses generated from this study can influence the design of interventional studies aimed at increasing awareness and educational programs in underserved communities. An overwhelming majority of participants attended one movement disorders clinic, under the care of one provider, limiting generalizability. This site was selected because of its location in downtown Atlanta, with easier access for ethnic minorities compared to other DBS implantation centers in the city [51]. Also, this study excluded non-English speakers and other minority individuals. Since language and culture can influence perceptions of procedures, future studies should include diverse underserved populations. Finally, objective symptom severity measures were not used due to incomplete data. Although this may introduce recall bias, the focus of the study was on patients’ perceptions, a powerful factor in decision-making in the context of DBS.

## Conclusion

Although participants were generally unwilling to undergo DBS in their current state, many expressed openness to DBS if their symptoms were inadequately controlled despite maximal medical therapy. This suggests that Black patients may be receptive to DBS when presented as a viable option to improve QoL. The results from this study reveal the impact that systemic barriers have on decreased DBS utilization rates, as opposed to personal unwillingness. Possible barriers include limited DBS awareness, decreased avenues of educational access in underserved populations, and implicit bias in provider decision making. To address some of these barriers, clinicians should begin longitudinal DBS discussions early and encourage patients to speak with others that have undergone the procedure. This can be facilitated through connections with peer advocates or increased participation in support groups or PD-specific community programs.

## Supplementary Material

Supplementary materials

Supplementary material associated with this article can be found, in the online version, at https://doi.org/10.1016/j.neuros.2025.100012.

## Figures and Tables

**Table 1:** Patient Demographics and Survey Responses.

Variables	Median [IQR] or N (%)
Age, in years (n = 28)	73.5 [64.5, 75.0]
Male Sex	14 (50)
Level of Education	
High School	14 (50)
Bachelor’s Degree	9 (32)
Post-Graduate Degree	5 (18)
Time from Diagnosis, in months	24 [8.5, 36]
Perceived Disability, Likert scale	4 [3, 4.5]
Most Bothersome Symptom	
Bradykinesia	2 (7)
Tremor	13 (46)
Walking	8 (29)
Other	5 (18)
Number of Different Medications	1.0 [0.5. 1.5]
Medication Frequency	3 [1.5, 3.5]
Awareness of Other Treatment Options	
Yes	9 (32)
No	19 (68)
Awareness of DBS	
Yes	9 (32)
No	19 (68)
Would you Undergo DBS Today?	
Yes	8 (29)
No	14 (50)
Maybe	6 (21)
Would you Consider DBS in the Future?	
Yes	19 (68)
No	4 (14)
Maybe	5 (18)

Abbreviations: DBS, deep brain stimulation; IQR, interquartile range

**Table 2:** Factors Associated with Prior DBS Awareness.

	Yes (n = 9)	No/Maybe (n = 19)	p- value
Age, Median [IQR]	73 [67, 75]	68 [63, 73]	0.22
Sex			1
Male	4 (44)	10 (53)	
Female	5 (56)	9 (47)	
Level of Education, n (%)			0.42
High School	4 (45)	10 (53)	
Bachelor’s Degree	2 (22)	7 (37)	
Post-Graduate Degree	3 (33)	2 (10)	
Time from Diagnosis in months, Median [IQR]	56 [92]	36 [71]	0.49
Perceived Disability (Likert Scale), Median [IQR]	3 [2, 4]	3 [3, 4]	0.80
Most Bothersome Symptom, n (%)			1
Bradykinesia	0 (0)	2 (11)	
Tremor	4 (45)	9 (47)	
Walking	3 (33)	5 (26)	
Other	2 (22)	3 (16)	
Number of Different Medications, Median [IQR]	2 [1, 2]	1 [1, 2]	0.62
Medication Frequency, Median [IQR]	3 [3, 3]	3 [2, 3]	0.96
Awareness of Other Treatment Options, n (%)			0.01*
Yes	6 (67)	3 (16)	
No	3 (33)	16 (84)	

*P*-values were based on Fisher’s exact test for categorical variables and Wilcoxon rank-sum test for continuous variables.

(*) Asterix indicates significance at p < 0.05.

**Table 3:** Factors Associated with Current Willingness to Undergo DBS.

	Yes (n = 8)	No/Maybe (n = 20)	p- value
Age, Median [IQR]	68 [62, 72]	72 [65, 75]	0.23
Sex			0.03*
Male	7 (87)	7(35)	
Female	1 (13)	13 (65)	
Level of Education, n (%)			0.19
High School	6 (75)	8 (40)	
Bachelor’s Degree	2 (25)	7 (35)	
Post-Graduate Degree	0 (0)	5 (25)	
Time from Diagnosis in months, Median [IQR]	58 [39, 97]	36 [18, 68]	0.13
Perceived Disability, Likert scale, Median [IQR]	5 [4, 5]	3 [2, 4]	0.03*
Most Bothersome Symptom, n (%)			0.09
Bradykinesia	1 (12)	1 (5)	
Tremor	6 (75)	7 (35)	
Walking	0 (0)	8 (40)	
Other	1 (13)	4 (20)	
Number of Different Medications, Median [IQR]	2 [2, 2]	1 [0, 2]	0.04*
Medication Frequency, Median [IQR]	3 [3, 3]	3 [0, 4]	0.45
Awareness of Other Treatment Options, n (%)			1
Yes	2 (25)	7 (35)	
No	6 (75)	13 (65)	

*P*-values were based on Fisher’s exact test for categorical variables and Wilcoxon rank-sum test for continuous variables.

(*) Asterix indicates significance at p < 0.05.

**Table 4:** Factors Associated with Future Willingness to Undergo DBS.

	Yes (n = 19)	No/Maybe (n = 9)	p- value
Age, Median [IQR]	67 [63, 74]	72 [68, 83]	0.23
Sex			0.10
Male	12 (63)	2(22)	
Female	7 (37)	7 (78)	
Level of Education, n (%)			1
High School	9 (47)	5 (56)	
Bachelor’s Degree	6 (32)	3 (33)	
Post-Graduate Degree	4 (21)	1 (11)	
Time from Diagnosis in months, Median [IQR]	60 [18, 87]	36 [19, 36]	0.05
Perceived Disability (Likert scale), Median [IQR]	4 [3, 4]	3 [2, 4]	0.53
Most Bothersome Symptom, n (%)			0.06
Bradykinesia	0 (0)	2 (22)	
Tremor	11 (58)	2 (22)	
Walking	4 (21)	4 (45)	
Other	4 (21)	1 (11)	
Number of Different Medications, Median [IQR]	2 [1, 2]	0 [0, 1]	0.002*
Medication Frequency, Median [IQR]	3 [3, 4]	0 [0, 3]	0.01*
Awareness of Other Treatment Options, n (%)			0.67
Yes	7 (37)	2 (22)	
No	12 (63)	7 (78)	

*P*-values were based on Fisher’s exact test for categorical variables and Wilcoxon rank-sum test for continuous variables.

(*) Asterix indicates significance at p < 0.05.

## Glossary

DBS: deep brain stimulation
REM: rapid eye movement
SES: socioeconomic status
PD: Parkinson’s disease
QoL: quality of life
