# Supplementary material for: Exploring deep brain stimulation willingness in Black individuals with Parkinson’s disease
Source: Equity Neurosci. Author manuscript; Available in PMC 2026 Jan 14. (PMC12799251; doi:10.1016/j.neuros.2025.100012)
Supplement: Supplementary materials [file NIHMS2114646-supplement-Supplementary_materials.docx]

**Supplementary Figure 1: DBS Willingness and Perceptions Survey**

Hello, my name is *[insert name here*]. I am one of the [*residents/medical students]* at [*Emory/Morehouse University School of Medicine*] that works closely with the neurology clinic. Sorry to take up some of your time, but our schools are doing a survey to better understand people’s awareness of advanced therapies for Parkinson’s disease. Specifically, we are looking for people that identify as African American or Black who have a diagnosis of Parkinson’s disease, who are willing to take a survey to better gauge a sense of their understanding of deep brain stimulation and their theoretical willingness to undergo that procedure.

1. What month and year were you diagnosed with Parkinson’s disease?
2. On a scale of 1 to 5, with 5 being extremely disabling, how bad would you say the symptoms of Parkinson’s affect you on a daily basis?

[ ] 1 = barely disabling/not disabling at all

[ ] 2 = slightly disabling

[ ] 3 = moderately disabling

[ ] 4 = very disabling

[ ] 5 = extremely disabling

1. What symptom, if any, is the most troublesome?
2. How many times a day are you taking your medications for Parkinson’s disease?
3. How many different medications are you taking for Parkinson’s disease?
4. Has anyone ever talked to you about another option for treatment? If so, which ones?
5. Have you heard of deep brain stimulation as a treatment for Parkinson’s disease?

Explain to them briefly what DBS is and possible side effects and benefits:

Deep Brain stimulation is a surgical procedure where they implant electrodes in a specific part of your brain by a skilled neurosurgeon. You will also have an implantable pacemaker like device in your chest that will allow you to control the amount of stimulation that goes through your head. It is used commonly in Parkinson’s patients who have bad tremors and significant slowness *or stiffness* that is not well controlled with current medications. It is a low-risk procedure, but it does come with some possible risk such as seizure, headache, cognitive problems, infection, stroke. The good thing about this procedure is that it is fully covered under all insurance types and is also a reversible procedure.

1. Given the information and your current symptoms, would you consider undergoing DBS as a treatment option?   Yes/ No/ Maybe
2. Why? _________________________________
3. Would you ever consider DBS as a possible treatment if your symptoms were still disabling while on maximum medication management? Yes/ No/ Maybe
4. Why? _________________________________

**Supplementary Table 1: Factors Associated with Current Willingness to Undergo DBS (YES/MAYBE vs NO)**

|  | **Yes/Maybe**  **(n = 14)** | **No**  **(n = 14)** | **p- value** |
| --- | --- | --- | --- |
| Age, Median [IQR] | 65 [63, 70] | 73[68, 75] | **0.02*** |
| Sex  Male  Female | 9 (64)  5 (36) | 5 (36)  9 (64) | 0.13 |
| Level of Education, n (%)  High School  Bachelor’s Degree  Post-Graduate Degree | 10 (71)  4 (29)  0 (0) | 4 (28)  5 (36)  5 (36) | **0.03*** |
| Time from Diagnosis (months),, Median [IQR] | 49 [30, 87] | 36 [18, 65] | 0.30 |
| Perceived Disability (Likert scale), Median [IQR] | 4 [3, 5] | 3 [2, 3] | **0.03*** |
| Most Bothersome Symptom, n (%)  Bradykinesia  Tremor  Walking  Other | 1 (7)  8 (57)  4 (29)  1 (7) | 1 (7)  5 (35)  4 (29)  4 (29) | 0.51 |
| Number of Different Medications, Median [IQR] | 2 [1, 2] | 1 [0, 2] | 0.09 |
| Medication Frequency, Median [IQR] | 3 [3, 3] | 3 [0, 3] | 0.09 |
| Awareness of Other Treatment Options, n (%)  Yes  No | 5 (36)  9 (64) | 4 (29)  10 (71) | 1 |

*P*-values were based on Fisher’s exact test for categorical variables and Wilcoxon rank-sum test for continuous variables. Asterix (*) indicates significance at p < 0.05.

**Supplementary Table 2: Factors Associated with Future Willingness to Undergo DBS (YES/MAYBE vs NO)**

|  | **Yes/Maybe**  **(n = 24)** | **No**  **(n = 4)** | **p- value** |
| --- | --- | --- | --- |
| Age, Median [IQR] | 68 [63, 74 | 79[73, 86] | **0.03*** |
| Sex  Male  Female | 13 (54)  11 (46) | 1 (25)  3 (75) | 0.60 |
| Level of Education, n (%)  High School  Bachelor’s Degree  Post-Graduate Degree | 12 (50)  7 (29)  5 (21) | 2(50)  2 (50)  0 (0) | 0.80 |
| Time from Diagnosis (months), Median [IQR] | 49 [19, 75] | 28 [12, 36] | 0.15 |
| Perceived Disability (Likert scale), Median [IQR] | 3 [3, 4] | 3 [2, 5] | 0.89 |
| Most Bothersome Symptom, n (%)  Bradykinesia  Tremor  Walking  Other | 2 (8)  12 (50)  6 (25)  4 (17) | 0 (0)  1 (25)  2 (50)  1 (25) | 0.63 |
| Number of Different Medications, Median [IQR] | 2 [1, 2] | 0 [0, 1] | **0.01*** |
| Medication Frequency, Median [IQR] | 3 [3, 4] | 0 [0, 2] | **0.01*** |
| Awareness of Other Treatment Options, n (%)  Yes  No | 8 (33)  16 (67) | 1 (25)  3 (75) | 1.0 |

*P*-values were based on Fisher’s exact test for categorical variables and Wilcoxon rank-sum test for continuous variables. Asterix (*) indicates significance at p < 0.05.

**Supplementary Table 3: Factors Associated with Current Willingness to Undergo DBS (YES vs NO)**

|  | **Yes**  **(n = 8)** | **No**  **(n = 14)** | **p- value** |
| --- | --- | --- | --- |
| Age, Median [IQR] | 68 [62, 72] | 73[68, 75] | 0.15 |
| Sex  Male  Female | 7 (87)  1 (13) | 5 (36)  9 (64) | **0.03*** |
| Level of Education, n (%)  High School  Bachelor’s Degree  Post-Graduate Degree | 6 (75)  2 (25)  0 (0) | 4 (28)  5 (36)  5 (36) | 0.09 |
| Time from Diagnosis (months), Median [IQR] | 58 [39, 97] | 36 [18, 65] | 0.17 |
| Perceived Disability (Likert scale), Median [IQR] | 5 [4, 5] | 3 [2, 3] | **0.02*** |
| Most Bothersome Symptom, n (%)  Bradykinesia  Tremor  Walking  Other | 1 (12)  6 (75)  0 (0)  1 (13) | 1 (7)  5 (35)  4 (29)  4 (29) | 0.22 |
| Number of Different Medications, Median [IQR] | 2 [2, 2] | 1 [0, 2] | 0.05 |
| Medication Frequency, Median [IQR] | 3 [3, 3] | 3 [0, 3] | 0.17 |
| Awareness of Other Treatment Options, n (%)  Yes  No | 2 (25)  6 (75) | 4 (29)  10 (71) | 1 |

*P*-values were based on Fisher’s exact test for categorical variables and Wilcoxon rank-sum test for continuous variables. Asterix (*) indicates significance at p < 0.05.

**Supplementary Table 4: Factors Associated with Future Willingness to Undergo DBS (YES vs NO)**

|  | **Yes**  **(n = 19)** | **No**  **(n = 4)** | **p- value** |
| --- | --- | --- | --- |
| Age, Median [IQR] | 67 [63, 74] | 79 [73, 86] | **0.04*** |
| Sex  Male  Female | 12 (63)  7 (37) | 1 (25)  3 (75) | 0.28 |
| Level of Education, n (%)  High School  Bachelor’s Degree  Post-Graduate Degree | 9 (47)  6 (32)  4 (21) | 2(50)  2 (50)  0 (0) | 0.80 |
| Time from Diagnosis (months), Median [IQR] | 60 [18, 87] | 28 [12, 36] | 0.11 |
| Perceived Disability (Likert scale), Median [IQR] | 4 [3, 4] | 3 [2, 5] | 0.83 |
| Most Bothersome Symptom, n (%)  Bradykinesia  Tremor  Walking  Other | 0 (0)  11 (58)  4 (21)  4 (21) | 0 (0)  1 (25)  2 (50)  1 (25) | 0.39 |
| Number of Different Medications, Median [IQR] | 2 [1, 2] | 0 [0, 1] | **0.01*** |
| Medication Frequency, Median [IQR] | 3 [3, 4] | 0 [0, 2] | **0.01*** |
| Awareness of Other Treatment Options, n (%)  Yes  No | 7 (37)  12 (63) | 1 (25)  3 (75) | 1.0 |

*P*-values were based on Fisher’s exact test for categorical variables and Wilcoxon rank-sum test for continuous variables. Asterix (*) indicates significance at p < 0.05.
